# Trends in Healthcare Resource Use Preceding Diagnosis of Alzheimer's Disease Dementia

**DOI:** 10.1155/2023/8154701

**Published:** 2023-01-07

**Authors:** Ibrahim M. Abbass, Dae Choi, Christopher Wallick, Sheila Seleri Assunção

**Affiliations:** Genentech, A Member of the Roche Group, South San Francisco, CA, USA

## Abstract

**Introduction:**

An Alzheimer's disease (AD) dementia diagnosis is often preceded by an extended period of cognitive decline. Few studies have examined healthcare resource use (HRU) during an extended period before AD dementia diagnosis.

**Methods:**

In a historical claims-based cohort study, propensity score-matched cohorts of patients with and without AD dementia were observed for a 5-year prediagnosis period and a 1-year postdiagnosis period. Demographics, clinical characteristics, and HRU were compared between groups.

**Results:**

Individuals in the AD dementia group displayed a greater level of medical complexity in the year before diagnosis of AD dementia relative to those in the matched cohort. Both all-cause and AD dementia complication-related HRU increased gradually, with a marked spike at the time of initial AD dementia diagnosis. *Discussion*. Further research into the natural history of patients with AD dementia is necessary to improve identification of early AD and to better understand its broader impact.

## 1. Background

Alzheimer's disease (AD) dementia is estimated to affect approximately 5.5 million Americans, and an additional 2.43 million are estimated to have mild cognitive impairment (MCI) due to AD [1]; the vast majority of these individuals are 65 years of age and older [2]. The number of people age 65 and older with AD dementia is projected to reach 12.7 million by the year 2050 [2]. Using a model that incorporated AD biomarkers, Brookmeyer et al. [1] estimated that the prevalence of MCI due to AD is also expected to grow to 5.7 million by 2060. The demands on healthcare resource use (HRU) and associated costs are anticipated to increase in parallel, with healthcare costs increasing to $1.1 trillion in 2050 [2].

Diagnosis of AD dementia is often preceded by an extended period characterized primarily by decline in cognitive function and memory, namely, MCI due to AD. However, most research describing treatment patterns and costs in AD has focused on individuals in the dementia phase; few have examined HRU during the MCI due to AD period. Albert et al. observed increased use of ambulatory and outpatient care among medicare beneficiaries in the 2 years preceding AD dementia diagnosis and estimated excess costs of 26% for women and 85% for men [3]. Ramakers et al. [4] and Eisele et al. [5] found that general practitioner contact frequency was elevated among individuals ultimately diagnosed with AD dementia. Suehs et al. [6] found that healthcare expenditures—and specifically, medical costs—were also elevated in the year before diagnosis of AD dementia among Medicare Advantage beneficiaries. Finally, recent work conducted in a Medicare Advantage population showed greater HRU and healthcare costs in persons diagnosed with AD dementia during the 6-month period immediately preceding the diagnosis, compared with other prediagnosis timeframes [7].

The goals of this study were to (1) describe the demographic and clinical characteristics for individuals who were newly diagnosed with AD dementia and for a propensity-matched cohort of individuals with no evidence of AD dementia (referred to as the non-AD comparison group in this manuscript) and (2) examine pre- and postdiagnosis patterns of HRU associated with persons with AD dementia and for a non-AD comparison group. This study advances the current understanding of AD dementia by building on previous research, including expanding the window of observation to 5 years before a confirmatory AD dementia diagnosis and examining a wider variety of clinical, socioeconomic, and behavioral characteristics than prior studies. Findings of this work will improve understanding of the period preceding AD dementia diagnosis and help identify potentially unmet medical needs and opportunities for timely diagnosis and intervention earlier in the AD continuum.

## 2. Methods

This was a historical cohort study using administrative claims data. Individuals with newly diagnosed AD dementia were identified and observed for a 5-year period: prediagnosis through 1 year postdiagnosis of AD dementia. We compared demographic and clinical characteristics, as well as HRU, in individuals who developed AD dementia and in a matched cohort of individuals who were not diagnosed with AD dementia during a similar period of time.

### 2.1. Data Sources

The data source for this study was a large US-based administrative claims database. This database contains enrollment information linked to medical, laboratory, and pharmacy claims data for Medicare Advantage members. Supplementary socioeconomic information typically unavailable in administrative claims data (e.g., education level) was obtained from a third-party proprietary commercial data source that was linked at individual member level to the administrative claims data. The study protocol was reviewed and approved by an external institutional review board before study initiation.

### 2.2. Study Population

Individuals with newly diagnosed AD dementia were identified using ICD-9 and ICD-10 diagnosis codes (ICD-9 CM: 331.0; ICD-10 CM: G30.X) reported in medical claims records on at least 2 separate service dates between 1 January 2012 and 30 June 2018. The date of the first observed diagnosis code was defined as the index event. A comparison group consisting of individuals with no evidence of AD dementia was constructed via propensity score matching (described in Section 2.4.1) to the AD dementia cohort using a series of year-specific subject identification and matching procedures. Candidates for the propensity-matched non-AD cohort were required to have evidence of seeking medical care on 2 separate services dates within the same calendar year. Each calendar year was examined separately and the index date for the non-AD group was the date of first medical claim. To be included in the non-AD comparison group, individuals could have no evidence of a diagnosis of AD dementia, dementia of any other etiology, or a prescription claim for an acetylcholinesterase inhibitor and/or memantine at any point during their health plan enrollment. Inclusion criteria for both the AD dementia and the matched cohorts were ≥65 years of age at index, continuous enrollment in a Medicare Advantage Prescription Drug plan for ≥5 years before and for ≥1 year after the index date. After initial identification of persons eligible for inclusion, propensity score matching proceeded as described in Section 2.4(Statistical Analysis).

### 2.3. Measures

#### 2.3.1. Sociodemographic and Clinical Characteristics

Age, sex, geographic region of residence, and race/ethnicity were determined based on the Medicare enrollment file as of the index date. Population density was divided into three categories: rural, urban, and suburban and was assigned by matching patients' zip codes to rural-urban commuting area codes [8]. Insurance plan characteristics (i.e., low income subsidy and dual eligibility) were obtained from enrollment file information. Low income subsidy status includes individuals with limited resources and an income below 150% of the US federal poverty threshold who were eligible for additional premium and cost-share assistance for prescription drugs. Individuals in the dual eligibility category are eligible for both Medicare and Medicaid.

#### 2.3.2. Clinical Characteristics and Comorbidities

Clinical indices, including the Charlson Comorbidity Index (CCI), an index of Elixhauser comorbidities (ECs), and the RxRisk-V score (RRS) were calculated, along with a measure of frailty. The CCI and EC were calculated based on the presence of ICD-9 and ICD-10 diagnosis codes reported on inpatient and outpatient medical claims based on the implementation of Quan et al. and Elixhauser et al. [8, 9]. The CCI uses 17 categories of clinical conditions, with each condition assigned a weight from 1 to 6 (e.g., congestive heart failure, weight of 1; moderate/severe liver disease, weight of 3). The condition weights for the observed comorbidities for a given individual are summed to calculate the CCI score [10]. The EC includes 31 separate medical condition categories (e.g., uncomplicated diabetes and coagulopathy). Indicator variables were coded for each EC. In addition, a summary score was derived as the unweighted sum of the individual ECs present [11]. The RRS is a pharmacy-based comorbidity index that includes 45 distinct medical condition categories via their associated medication treatments [12]. The RRS is determined by summing the number of unique condition categories, with higher scores indicating greater comorbidity burden; it has been shown to be predictive of healthcare costs and mortality in a range of populations [11–16].

Frailty was assessed using the methods and codes described by Farout et al. [17]. Individuals with at least one frailty indicator were identified; the average number of frailty indicators and the number of individuals with any of the top 4 predictors of activities of daily living dependency (i.e., Parkinson's disease, paralysis, use of wheelchair, and use of home hospital bed) were reported.

#### 2.3.3. Healthcare Resource Use

The following HRU parameters were assessed: physician office visits, emergency department visits, inpatient admissions, home health use, nursing facility residency, and hospice use. To identify temporal trends, HRU was assessed in 3-month (i.e., quarterly) intervals over the pre- and postindex periods. Physician office visits, emergency department visits, and inpatient episodes of care were identified from provider and facility claims using bill type, revenue, and place of treatment codes as well as dates of service. Multiple inpatient episodes, in which the discharge and admission dates were within one day, were considered a transfer and were collapsed into a single inpatient episode. Emergency department visits that resulted in an inpatient admission were considered part of the subsequent inpatient admission. Home health use, nursing facility residency, and hospice use were identified based on medical claims and/or information in the member's enrollment file.

Cognitive condition-related service use was identified and included physician office visits, emergency department visits, and inpatient admissions where AD dementia or a cognitive condition was coded in the primary diagnosis code position (see Supplementary Table A.1 for codes). In order to assess complications potentially related to AD dementia, physician office visits, emergency department visits, and inpatient admissions related to the following medical complications commonly associated with AD were identified based on the presence of the diagnosis code in the primary position on the claim: skin ulcers, urinary tract infections, falls/fractures, malnutrition, and pneumonia (see Supplementary Table A.2 for codes) [18].

### 2.4. Statistical Analysis

#### 2.4.1. Propensity Score Matching

**Figure 1:**
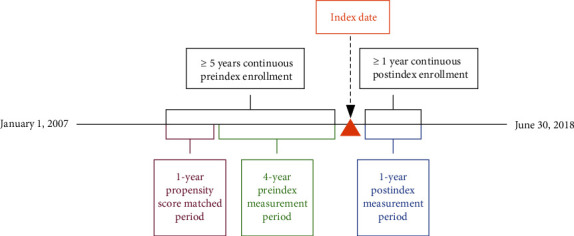
Study design schematic.

Propensity score matching is a statistical technique for generating comparison groups that are balanced across a large number of factors. It was used in this study to create a comparison group of individuals who had no evidence of developing AD dementia, but who had similar demographic and past clinical characteristics as those who ultimately developed AD dementia. A propensity score was estimated for each eligible AD dementia and non-AD cohort member by modeling the probability of a diagnosis of AD dementia conditional on a set of baseline characteristics. The propensity score was calculated using a set of prespecified demographic, clinical, and resource utilization variables measured during the first year of the 5-year preindex period (i.e., 49–60 months preceding the index event; Figure 1).

For the AD dementia cohort, individuals were identified and classified by calendar year according to their index date. Individuals eligible for inclusion in the non-AD cohort were assessed to determine whether or not they met the study inclusion criteria (2 healthcare encounters on separate dates) during any given calendar year of the study period. A total of 6 year-specific propensity score models were fit. To ensure an equal distribution by year of index across the 2 groups, the propensity score models were built sequentially, starting with calendar year 2012 and proceeding with each calendar year through 2017. Once a non-AD individual was matched to a patient with AD dementia, the patients were not eligible for matching in a subsequent year. The variables included in each year-specific propensity score model were the same and included the following: demographic characteristics (age, sex, race/ethnicity, low income status, dual eligibility status, geographic region, and month of index event), clinical characteristics (indicators for the EC and RRS categories), and HRU measures (number of physician office visits, number of emergency department visits, and number of inpatient hospitalizations). Matching was conducted at a 1 : 1 ratio using a greedy matching technique [19]. Univariate standardized differences and the c-statistic of the propensity score model run using only the matched pairs were examined to assess balance.

#### 2.4.2. Descriptive Analysis of Demographic and Clinical Characteristics for Individuals Newly Diagnosed with AD Dementia

Demographic and clinical characteristics were summarized for individuals newly diagnosed with AD dementia and compared with individuals without AD dementia. Medical conditions were measured using medical claims adjudicated during the 1-year period immediately preceding initial diagnosis of AD dementia (year 5 preindex). Descriptive statistics, including the number and percentage of individuals with each characteristic, were reported. Comparisons between the AD dementia and non-AD groups were based on chi-square tests for categorical variables.

#### 2.4.3. Pre- and Postdiagnosis Patterns of HRU Associated with AD and for a Non-AD Comparison Group

All-cause HRU was examined, along with patterns of HRU associated with AD dementia and AD dementia-related complications. Outpatient service types examined included physician office visits, outpatient visits, home health services, nursing facility residency, and hospice stay. Inpatient service types examined included inpatient admissions, emergency department visits, and skilled nursing facility admissions. Service use rates were calculated in quarterly intervals over the duration of the pre- and postindex period in order to identify trends in service use over time. The number of encounters for 1000 individuals was calculated for physician office visits, outpatient visits, emergency department visits, and skilled nursing facility admissions. The proportion of individuals with an encounter during the measurement period per 1000 individuals was calculated for home health services, hospice, and nursing facility residency. Quarterly service use rates were summarized descriptively for both cohorts.

All data analyses were conducted using SAS version 9.4. The a priori alpha level for all inferential analyses was set at 0.05; all statistical tests were two-tailed, unless otherwise specified.

## 3. Results

### 3.1. Cohort Construction and Propensity Score Matching

**Figure 2:**
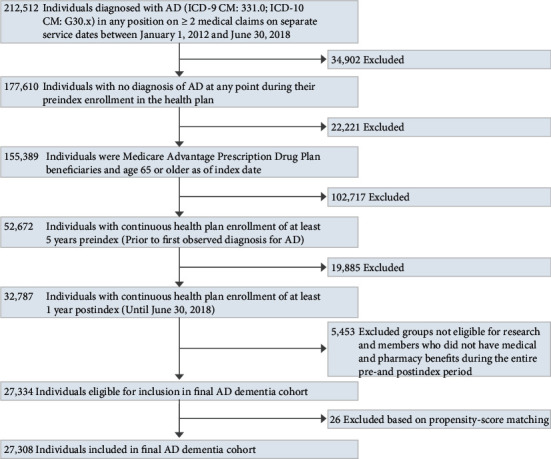
Attrition diagram for the AD dementia cohort.

A total of 27,334 individuals newly diagnosed with AD dementia met study selection and continuous enrollment criteria (Figure 2). More than 1.8 million eligible enrollment episodes without evidence of a diagnosis of AD dementia or medication treatment indicated for AD dementia were considered for propensity score matching. A total of 27,308 individuals (99.9%) in the AD dementia cohort were successfully matched to individuals in the non-AD comparison group. All standardized differences in the postmatch sample were <0.02 and the c-statistic for the propensity score model fit on the final matched sample was 0.518, indicating balance on a wide array of variables both individually and overall.

### 3.2. Demographic and Clinical Characteristics of Matched Cohorts

Baseline characteristics for the matched study cohort at the time of matching are reported in Table 1. Prevalence of Elixhauser comorbidities both at the time of matching (year 1 preindex) and as assessed in the 1-year period immediately prior to AD diagnosis (year 5 preindex) is reported in Table 2. Most individuals included in the matched cohorts were women (both groups: 63.6%) and white (AD dementia group: 81.8%; non-AD group: 82.1%). The cohort consisted primarily of urban (AD dementia group: 69.4%; non-AD group: 70.4%) and suburban-dwelling (AD dementia group: 20.8%; non-AD group: 18.8%) individuals. Persons in the AD dementia group had a greater number of comorbidities in year 5 preindex compared with the non-AD group, as reflected in the mean EC score (3.6 vs. 2.7, *P* < 0.001), the mean RRS (5.9 vs. 4.9, *P* < 0.001), and the mean CCI score (2.3 vs. 1.7, *P* < 0.001). In particular, cardiovascular disease, neurological conditions other than AD dementia, depression, and other psychiatric conditions were more common in the AD dementia group compared with the non-AD group. Similarly, every frailty indicator was more prevalent in the AD dementia group compared with the non-AD group; and the top 4 frailty indicators were 3.5 times as likely to be present (12.6% vs. 3.6%, *P* < 0.001). The most common frailty indicators among patients in the AD dementia group were cognitive conditions (75.5%), arthritis (52.3%), ambulance use (29.6%), difficulty walking (28.3%), and psychiatric conditions (28.0%; Table 1). Notably, both the individual EC and the RRS categories were included in the matching process; however, the clinical measures included in the propensity score model were measured only during year 1 of the 5-year preindex period. The clinical characteristics reported here are based on year 5 preindex. There is therefore a period of 3 years for clinical divergence to emerge between the AD dementia and non-AD groups, and it is these emergent differences that are reflected in this comparison.

### 3.3. All-Cause Healthcare Resource Use

**Figure 3:**
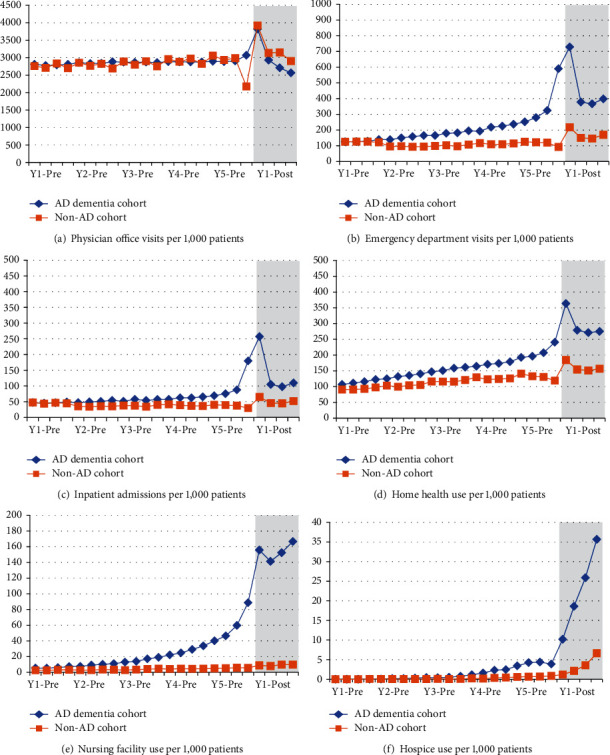
All-cause healthcare resource use, 5 years pre- and 1 year postindex. Grey shading indicates the 1 year postindex period.

Results from the analysis of all-cause HRU are shown in Figure 3 and Supplementary Table B.1. Healthcare resource use patterns among individuals who developed AD dementia reveal greater use of emergency department, inpatient, home health, nursing facility, and hospice use compared with those in the non-AD comparison group. In particular, there was substantial early divergence in rates of emergency department and home health service use starting 4 years before the AD dementia index date, with utilization peaking in the period when AD dementia was diagnosed, and returning to a stable trajectory during the postdiagnosis period. Divergence in nursing facility utilization between groups was substantial, but most pronounced in year 5 preindex. Hospice use also trended much higher in the AD dementia group relative to the non-AD group.

### 3.4. Complication-Related Healthcare Resource Use

**Figure 4:**
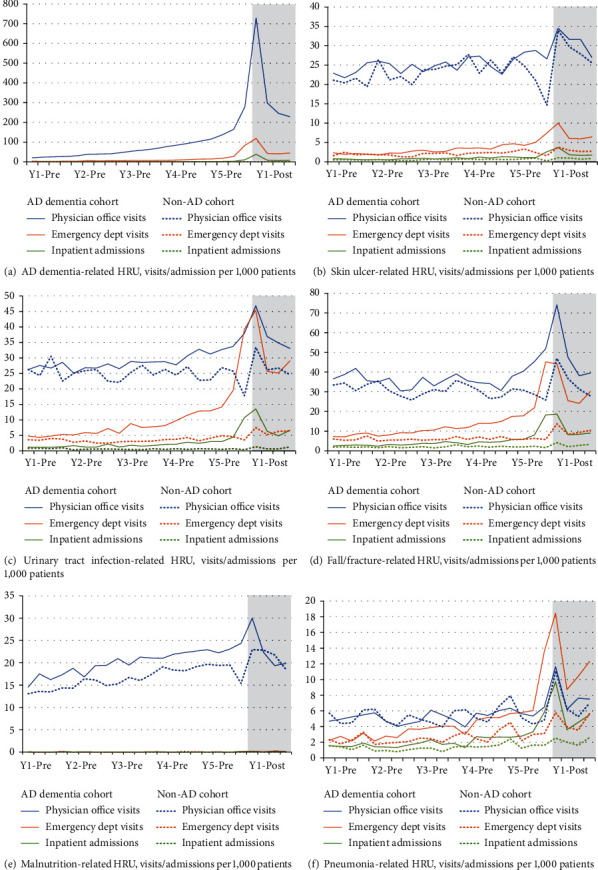
AD dementia complication-related healthcare resource use, 5 years pre- and 1 year postindex. Grey shading indicates the 1 year postindex period.

Results from analysis of the AD dementia complication-related HRU are shown in Figure 4 and see Supplementary Table C.1. Generally, rates of complication-related service use peaked at the time of indexing for both cohorts. Urinary tract infection and fall/fracture-related emergency department use, in particular, diverged early (approximately year 2, quarter 1 of the preindex period).

## 4. Discussion

Individuals diagnosed with AD dementia were more clinically complex than similar individuals who were not diagnosed with AD dementia. Despite robust propensity score matching at cohort entry (5 years prior to diagnosis of AD dementia), by the time the individuals were diagnosed with AD dementia, they had developed a greater number of comorbidities than individuals in the matched cohort. In particular, cardiovascular disease, neurological conditions, depression, and other psychiatric conditions were more common in the timeframe proximal to first diagnosis of AD dementia. Rates of diagnosis for conditions without a known association with AD dementia (e.g., cancers) were similar in both the AD dementia and non-AD groups, suggesting that these findings should not be attributed to use of nonexperimental methods.

Similarly, individuals diagnosed with AD dementia demonstrated a higher degree of frailty compared with individuals in the non-AD group. This result supports the findings of a recent systematic review by Borges et al. [20], which confirmed that frail older adults were at higher risk of incident cognitive disorders, especially vascular dementia, compared with nonfrail older adults. In our study, indicators of frailty were more common during the period immediately prior to the diagnosis of AD dementia (year 5 preindex). Individuals diagnosed with AD dementia also had patterns of engagement with the healthcare system that showed increased HRU years before their diagnosis of AD dementia. For these individuals, emergency department and home health service use were elevated early in the clinical course prior to diagnosis of AD dementia (year 1 preindex), and hospice use increased dramatically in the year after diagnosis of AD dementia. We hypothesize that similarly timed spikes in HRU in both cohorts from Q4 preindex to Q1 postindex trends may partially be attributable to healthcare-seeking behaviors of patients with progressive symptoms of AD. For example, a few months prior to AD diagnosis, patients may seek care in outpatient settings to understand the mental and/or behavioral symptoms they are experiencing. Some of these symptoms may lead to an emergency department visit or inpatient admission. Mental, behavioral, and neurodevelopmental symptoms are among the top 9 reasons for emergency department visits and hospital admission from the emergency department [21]. HRU spikes seen close to the time of diagnosis also may reflect hesitancy of healthcare providers to diagnose patients with AD at an early stage and tendency to formally diagnose AD only when symptoms progress and start to incur a higher risk or burden. Notably, patients residing in nursing facilities—who are under constant supervision by healthcare workers—and their AD diagnoses seem to diverge early on and at a constant pace compared to the control. We also hypothesize that the small number of patients who reside in hospice care are more likely to be formally diagnosed with AD. Due to AD and other comorbidities, once patients are diagnosed with AD, their tendency to stay in hospice care progressively increases over time.

Alzheimer's disease dementia—and cognitive decline in general—is thought to be significantly underdiagnosed [22–24]. Given the slow progression of AD dementia, a considerable amount of time may elapse between symptom onset and diagnosis, and this gap results in patients presenting at later stages in the disease course, when physical and mental health may have appreciably deteriorated. Thus, understanding trends and utilization patterns long before initial AD dementia diagnosis becomes increasingly relevant, particularly with patients from groups experiencing socioeconomic or other health-related disadvantage. This study observed trends in the diagnoses and HRU during the 5-year period preceding initial identification of AD dementia and found increased diagnoses related to complications as patients neared initial AD dementia diagnosis. This likely reflects increased use of services among individuals as their cognitive impairment worsened or as related medical needs arose. This may also suggest that a treating physician may be more likely to assign the diagnosis of AD dementia to a patient who is more severe/advanced and starting to manifest complications associated with AD dementia (e.g., falls/fractures).

In the era of imminent approval of multiple disease-modifying therapies for AD, opportunities to intervene will rely more and more on diagnosis during the early stages of the disease. Analysis of big data offers a means of learning about unmet needs of people with early stages of AD dementia; such analyses have the potential to address the need for care strategies that recognize and address the HRU costs associated with AD care [25]. Healthcare systems and payers with access to big data could also utilize techniques with higher predictive accuracy, such as artificial intelligence and machine learning, to identify patients in the early stages of AD and to inform policy and clinical decision-making. This approach has potential to save healthcare payers and system downstream costs incurred by higher HRU as documented in this study.

The results from this study should be interpreted in the context of its limitations. The results of claims-based research may have been influenced by missing data, potential errors in coding, and unmeasured factors, such as psychosocial variables and other clinical variables. Additionally, data in this study were obtained from a single claims-based data source and the results may not be generalizable to the overall US population, or to subpopulations within certain geographic regions of the US. Finally, although AD dementia is characterized by a long period of gradual cognitive decline, coding for AD dementia is limited to a single-diagnosis code. There remains an unmet need for more granular ICD-10 coding that incorporates various stages of the disease continuum or validated disease-severity algorithms to improve claims-based research methodology in AD dementia.

Our study found that HRU tended to increase gradually, with a marked spike at the initial AD dementia diagnosis, including both all-cause utilization (e.g., office visits, emergency department, and hospitalizations) and AD dementia complication-related utilization (e.g., falls/fractures, skin ulcers, and malnutrition). Improved understanding of the natural history and HRU of patients with AD dementia early in their disease progression is greatly needed and will be critically important as potential disease-modifying therapies emerge and are potentially used in early AD. Findings of this study shed light on patterns of care that foreshadow a diagnosis of AD dementia. This could guide interventions to diagnose and target care earlier in the disease process.

## Figures and Tables

**Table 1:** Baseline demographics and clinical characteristics, matched AD dementia and non-AD cohorts.

Baseline characteristics	Year 1 preindex (matching period)				Stand. Difference
	AD		Non-AD		
Sample size	27,308		27,308		
Age, mean (SD)	81.6 (6.6)		81.7 (6.9)		0.0152
Female sex, *N* (%)	17,368	63.6%	17,356	63.6%	0.0009
White race, *N* (%)	22,345	81.8%	22,413	82.1%	0.0065
South region, *N* (%)	18,275	66.9%	18,133	66.4%	0.0110
LIS status (at index), *N* (%)	5,594	20.5%	5,527	20.2%	0.0061
DE status (at index), *N* (%)	4,550	16.7%	4,534	16.6%	0.0016
Number of physician office visits, mean (SD)	11.2 (10.2)		11.0 (9.9)		0.0181
Number of inpatient hospitalizations, mean (SD)	0.2 (0.5)		0.2 (0.5)		0.0102
Number of emergency department encounters, mean (SD)	0.5 (1.2)		0.5 (1.5)		0.0148

Abbreviations: AD: Alzheimer's disease; LIS: low income subsidy; DE: dual eligible.

**Table 2:** Comorbid medical conditions at matching (year 1 preindex) and in the 1-year period immediately prior to first observed diagnosis of Alzheimer's disease (year 5 preindex).

	Year 1 preindex (matching period)				Year 5 preindex (AD diagnosis period)				*P ^a^*
	AD		Non-AD		AD		Non-AD		
	*N*	%	*N*	%	*N*	%	*N*	%	
Congestive heart failure	1,981	7.3%	1,968	7.2%	4,099	15.0%	2,874	10.5%	<0.001
Cardiac arrhythmia	3,427	12.5%	3,318	12.2%	6,305	23.1%	4,671	17.1%	<0.001
Valvular disease	1,550	5.7%	1,484	5.4%	2,845	10.4%	1,919	7.0%	<0.001
Pulmonary circulatory disorder	444	1.6%	428	1.6%	1,145	4.2%	919	3.4%	<0.001
Peripheral vascular disease	2,798	10.2%	2,715	9.9%	5,381	19.7%	4,514	16.5%	<0.001
Hypertension (uncomplicated)	15,804	57.9%	15,603	57.1%	18,864	69.1%	16,317	59.8%	<0.001
Hypertension (complicated)	2,285	8.4%	2,231	8.2%	4,877	17.9%	3,933	14.4%	<0.001
Paralysis	105	0.4%	100	0.4%	327	1.2%	73	0.3%	<0.001
Other neurological disorders	945	3.5%	335	1.2%	4,007	14.7%	366	1.3%	<0.001
Chronic obstructive pulm. disease	3,318	12.2%	3,300	12.1%	4,982	18.2%	4,422	16.2%	<0.001
Diabetes (uncomplicated)	6,350	23.3%	6,205	22.7%	6,983	25.6%	6,407	23.5%	<0.001
Diabetes (complicated)	2,401	8.8%	2,401	8.8%	4,366	16.0%	3,955	14.5%	<0.001
Hypothyroidism	3,819	14.0%	3,676	13.5%	5,506	20.2%	4,593	16.8%	<0.001
Renal failure	3,110	11.4%	3,071	11.2%	6,459	23.7%	6,104	22.4%	<0.001
Liver failure	271	1.0%	259	0.9%	486	1.8%	373	1.4%	<0.001
Peptic ulcer disease	122	0.4%	99	0.4%	241	0.9%	104	0.4%	<0.001
HIV/AIDS	<10		<10		<10		<10		
Lymphoma	110	0.4%	118	0.4%	201	0.7%	186	0.7%	0.444
Metastatic cancer	74	0.3%	62	0.2%	140	0.5%	174	0.6%	0.054
Solid tumor: No metastasis	1,624	5.9%	1,603	5.9%	1,831	6.7%	1,918	7.0%	0.141
Rheumatoid arthritis	847	3.1%	837	3.1%	1,072	3.9%	979	3.6%	0.036
Coagulopathy	366	1.3%	350	1.3%	902	3.3%	549	2.0%	<0.001
Obesity	767	2.8%	741	2.7%	1,281	4.7%	1,372	5.0%	0.070
Weight loss	493	1.8%	483	1.8%	1,615	5.9%	413	1.5%	<0.001
Fluid electrolyte disorders	1,523	5.6%	1,476	5.4%	4,033	14.8%	1,435	5.3%	<0.001
Blood loss anemia	142	0.5%	127	0.5%	294	1.1%	142	0.5%	<0.001
Deficiency anemia	915	3.4%	914	3.3%	1,682	6.2%	1,069	3.9%	<0.001
Alcohol abuse	163	0.6%	148	0.5%	329	1.2%	144	0.5%	<0.001
Drug abuse	248	0.9%	254	0.9%	516	1.9%	397	1.5%	<0.001
Psychoses	283	1.0%	234	0.9%	1,232	4.5%	120	0.4%	<0.001
Depression	2,369	8.7%	2,249	8.2%	5,394	19.8%	2,150	7.9%	<0.001

^a^ *P* value is from chi-square test for the preindex year 5 comparison between AD dementia and non-AD cohorts. Abbreviations: AD: Alzheimer's disease.

## Data Availability

The data used to support the findings of this study are available from the corresponding author upon request.

## Abbreviations

AD: Alzheimer's disease
CCI: Charlson Comorbidity Index
ECs: Elixhauser comorbidities
HRU: Healthcare resource use
MCI: Mild cognitive impairment
RRS: RxRisk-V score

## Additional Points

*Systematic Review*. Few studies have examined healthcare resource use (HRU) during an extended period before diagnosis of Alzheimer's disease (AD) dementia. This historical claims-based cohort study examines HRU related to AD dementia over a 5-year prediagnosis period and a 1-year postdiagnosis follow-up period. Demographics, clinical characteristics, and HRU were compared across propensity-matched cohorts of patients (*N* = 27,308 per group) with and without AD dementia. *Interpretation*. The present study advances the current understanding of AD dementia by building on previous research, including expanding the window of observation to 5 years before a confirmatory diagnosis of AD dementia and examining a wider variety of clinical, socioeconomic, and behavioral characteristics than prior studies. *Future Directions*. Findings of this study shed light on patterns of care that foreshadow a diagnosis of AD dementia. This could guide interventions to diagnose and target care earlier in the disease process. *Highlights*. (i) This claims-based-matched cohort study shows HRU trends pre- and post-AD diagnosis. (ii) Pre-AD diagnosis: all-cause HRU is greater in patients with AD than without AD. (iii) AD complication-related HRU peaks at the time of AD dementia diagnosis. (iv) This study sheds light on unmet medical needs preceding diagnosis of AD dementia.

## Supplementary Materials

Supplementary Materials Supplementary Table A.1: the primary diagnosis codes used in this study. Supplementary Table A.2: the diagnosis codes for complications identified in this study. Supplementary Table B.1: the pre- and postdiagnosis patterns for all-cause healthcare resource use in both the AD dementia and non-AD cohorts. Supplementary Table C.1: the pre- and postdiagnosis patterns for all-cause healthcare resource use associated with AD dementia and AD dementia-related complications in both the AD dementia and non-AD cohorts.
