# Supplementary material for: Trends in Healthcare Resource Use Preceding Diagnosis of Alzheimer's Disease Dementia
Source: Int J Alzheimers Dis. 2023 Jan 7;2023:8154701. doi: 10.1155/2023/8154701 (PMC9840544; doi:10.1155/2023/8154701)
Supplement: Supplementary Materials — Supplementary Table A.1: the primary diagnosis codes used in this study. Supplementary Table A.2: the diagnosis codes for complications identified in this study. Supplementary Table B.1: the pre- and postdiagnosis patterns for all-cause healthcare resource use in both the AD dementia and non-AD cohorts. Supplementary Table C.1: the pre- and postdiagnosis patterns for all-cause healthcare resource use associated with AD dementia and AD dementia-related complications in both the AD dementia and non-AD cohorts. [file 8154701.f1.docx]

SUPPLEMENTARY TABLES

**Table A.1.** Alzheimer’s disease, dementia, and other cognitive condition diagnosis codes.

| **Diagnostic category** | **Diagnosis codes** | |
| --- | --- | --- |
|  | **ICD-9** | **ICD-10** |
| Alzheimer's disease | 331.0 | G30.X |
| Dementia with Lewy bodies | 331.82 | G31.83 |
| Pick's disease | 331.11 | G31.01 |
| Other frontotemporal dementia | 331.19 | G31.09 |
| Senile degeneration of brain | 331.2 | G31.1 |
| Cerebral degeneration in diseases classified elsewhere | 331.7 | G13.8, G31.2, G94 |
| Presenile or senile dementia | 290.0X, 290.1X, 290.2X, 290.3X, 290.8X, 290.9X | F03.9X |
| Vascular dementia | 290.4X | F01.5X |
| Amnestic disorder in conditions classified elsewhere | 294.0 | F04 |
| Dementia in conditions classified elsewhere without behavioral disturbance | 294.1X | F02.8X |
| Dementia, unspecified | 294.2X | F03.9X |
| Other persistent mental disorders due to conditions classified elsewhere | 294.8 | F06.1, F06.8 |
| Senility without mention of psychosis/ Age-related cognitive decline | 797 | R41.81, R54 |
| Memory loss/Disorientation | 780.93 | R41.1, R41.2, R41.3, R41.4 |
| Mild cognitive impairment | 331.83 | G31.84 |
| Altered mental status | 780.97 | R41.0 R41.82 |
| Cerebral degeneration | 331.9 | G31.9 |
| Other symptoms and signs involving cognitive functions | 799.5X | R41.8X, R41.9 |

**Table A.2.** Complications associated with Alzheimer’s disease and associated Clinical Classifications Software (CCS) categories.

| **Diagnostic category** | **CCS multilevel category** |
| --- | --- |
| Skin ulcers | 12.1 (skin and subcutaneous tissue infections) and 12.3 (chronic ulcer of skin) |
| Urinary tract infections | 10.1.4 (urinary tract infection) |
| Falls/fractures | 16.1 (trauma related joint disorders and dislocations) 16.2 (fractures) |
| Malnutrition | 3.5 (nutritional deficiencies) |
| Pneumonia | 8.1.1 (pneumonia) |

**Table B.1.** Pre- and post-diagnosis patterns of all-cause healthcare resource use for matched AD dementia
and non-AD cohorts.

| **AD dementia cohort (N = 27,308)** | | | | | | | | |
| --- | --- | --- | --- | --- | --- | --- | --- | --- |
| **HRU per 1,000 patients** | **Year 1 Pre-index** | | | | **Year 2 Pre-index** | | | |
|  | **Q1** | **Q2** | **Q3** | **Q4** | **Q1** | **Q2** | **Q3** | **Q4** |
| **All-cause outpatient services** |  |  |  |  |  |  |  |  |
| Physician office visits | 2,810.35 | 2,773.80 | 2,789.15 | 2,809.32 | 2,857.55 | 2,832.80 | 2,837.78 | 2,882.78 |
| Outpatient visits | 3,294.75 | 3,251.17 | 3,286.66 | 3,335.65 | 3,386.63 | 3,357.18 | 3,383.66 | 3,431.45 |
| Home health services^a^ | 106.96 | 111.21 | 115.68 | 122.53 | 125.05 | 131.90 | 135.38 | 140.40 |
| Hospice^a^ | 0.00 | 0.00 | 0.00 | 0.00 | 0.04 | 0.18 | 0.18 | 0.29 |
| Nursing facility residency^a^ | 5.46 | 5.38 | 6.01 | 7.36 | 7.51 | 9.19 | 10.25 | 10.99 |
| **All-cause inpatient services** |  |  |  |  |  |  |  |  |
| Inpatient admissions | 47.24 | 45.22 | 46.43 | 48.78 | 47.06 | 49.36 | 50.64 | 54.16 |
| Emergency department visits | 125.09 | 126.41 | 128.53 | 140.07 | 139.01 | 149.96 | 158.01 | 165.48 |
| Skilled nursing facility admissions | 5.35 | 6.26 | 6.08 | 7.87 | 6.44 | 7.10 | 7.84 | 8.50 |
| **Non-AD cohort (N = 27,308)** | | | | | | | | |
| **HRU per 1,000 patients** | **Year 1 Pre-index** | | | | **Year 2 Pre-index** | | | |
|  | **Q1** | **Q2** | **Q3** | **Q4** | **Q1** | **Q2** | **Q3** | **Q4** |
| **All-cause outpatient services** |  |  |  |  |  |  |  |  |
| Physician office visits | 2,758.97 | 2,707.92 | 2,833.46 | 2,700.78 | 2,854.80 | 2,766.77 | 2,823.90 | 2,690.46 |
| Outpatient visits | 3,233.96 | 3,192.18 | 3,332.28 | 3,172.51 | 3,343.20 | 3,240.66 | 3,314.89 | 3,171.93 |
| Home health services^a^ | 90.74 | 90.67 | 92.79 | 97.33 | 103.27 | 99.57 | 104.04 | 105.28 |
| Hospice^a^ | 0.00 | 0.00 | 0.00 | 0.07 | 0.07 | 0.04 | 0.04 | 0.04 |
| Nursing facility residency^a^ | 2.60 | 2.53 | 3.11 | 3.15 | 2.78 | 2.75 | 3.55 | 3.26 |
| **All-cause inpatient services** |  |  |  |  |  |  |  |  |
| Inpatient admissions | 47.28 | 43.72 | 46.43 | 44.82 | 35.19 | 33.8 | 35.59 | 35.01 |
| Emergency department visits | 125.75 | 126.26 | 126.19 | 122.31 | 96.02 | 97.11 | 93.64 | 94.11 |
| Skilled nursing facility admissions | 4.54 | 5.24 | 4.83 | 5.42 | 3.85 | 4.36 | 4.50 | 3.99 |

Unless otherwise stated, “HRU” reflects the number of events per 1,000 patients.

^a^Rates for home health services, hospice, and nursing facility residency reflect the number of patients per 1,000 (ie, proportion of patients).

**Abbreviations:** AD, Alzheimer’s disease; HRU, healthcare resource use.

**Table B.1.** Pre- and post-diagnosis patterns of all-cause healthcare resource use for matched AD dementia
and non-AD cohorts (cont’d).

| **AD dementia cohort (N = 27,308)** | | | | | | | | |
| --- | --- | --- | --- | --- | --- | --- | --- | --- |
| **HRU per 1,000 patients** | **Year 3 Pre-index** | | | | **Year 4 Pre-index** | | | |
|  | **Q1** | **Q2** | **Q3** | **Q4** | **Q1** | **Q2** | **Q3** | **Q4** |
| **All-cause outpatient services** |  |  |  |  |  |  |  |  |
| Physician office visits | 2,871.98 | 2,866.56 | 2,872.71 | 2,859.93 | 2,885.56 | 2,879.45 | 2,877.87 | 2,877.84 |
| Outpatient visits | 3,444.89 | 3,456.13 | 3,462.90 | 3,455.65 | 3,509.85 | 3,516.63 | 3,533.80 | 3,543.06 |
| Home health services^a^ | 146.92 | 150.65 | 158.63 | 161.31 | 164.35 | 170.76 | 173.43 | 178.74 |
| Hospice^a^ | 0.44 | 0.40 | 0.51 | 0.77 | 1.14 | 1.61 | 2.31 | 2.45 |
| Nursing facility residency^a^ | 13.04 | 13.95 | 17.17 | 19.04 | 22.19 | 24.75 | 29.15 | 33.73 |
| **All-cause inpatient services** |  |  |  |  |  |  |  |  |
| Inpatient admissions | 51.23 | 57.42 | 53.9 | 57.09 | 57.49 | 62.33 | 62.11 | 65.37 |
| Emergency department visits | 165.37 | 179.36 | 181.78 | 195.22 | 192.73 | 218.29 | 224.88 | 237.07 |
| Skilled nursing facility admissions | 9.45 | 10.77 | 10.62 | 11.64 | 11.39 | 13.95 | 14.98 | 16.30 |
| **Non-AD cohort (N = 27,308)** | | | | | | | | |
| **HRU per 1,000 patients** | **Year 3 Pre-index** | | | | **Year 4 Pre-index** | | | |
|  | **Q1** | **Q2** | **Q3** | **Q4** | **Q1** | **Q2** | **Q3** | **Q4** |
| **All-cause outpatient services** |  |  |  |  |  |  |  |  |
| Physician office visits | 2,891.24 | 2,797.82 | 2,898.20 | 2,751.68 | 2,958.18 | 2,882.09 | 2,974.37 | 2,824.59 |
| Outpatient visits | 3,408.52 | 3,308.74 | 3,435.51 | 3,260.91 | 3,494.25 | 3,431.78 | 3,533.84 | 3,360.96 |
| Home health services^a^ | 116.12 | 115.86 | 115.42 | 120.81 | 129.63 | 122.93 | 123.85 | 125.93 |
| Hospice^a^ | 0.04 | 0.11 | 0.04 | 0.07 | 0.22 | 0.18 | 0.37 | 0.44 |
| Nursing facility residency^a^ | 2.78 | 3.41 | 4.14 | 4.54 | 4.25 | 4.50 | 4.36 | 4.50 |
| **All-cause inpatient services** |  |  |  |  |  |  |  |  |
| Inpatient admissions | 37.83 | 37.28 | 34.13 | 39.66 | 41.42 | 38.71 | 36.69 | 36.44 |
| Emergency department visits | 99.02 | 103.30 | 96.97 | 107.07 | 117.00 | 108.91 | 109.16 | 115.09 |
| Skilled nursing facility admissions | 5.05 | 4.54 | 5.09 | 6.74 | 5.86 | 5.16 | 4.94 | 4.87 |

Unless otherwise stated, “HRU” reflects the number of events per 1,000 patients.

^a^Rates for home health services, hospice, and nursing facility residency reflect the number of patients per 1,000 (ie, proportion of patients).

**Abbreviations:** AD, Alzheimer’s disease; HRU, healthcare resource use.

**Table B.1.** Pre- and post-diagnosis patterns of all-cause healthcare resource use for matched AD dementia
and non-AD cohorts (cont’d).

| **AD dementia cohort (N = 27,308)** | | | | | | | | |
| --- | --- | --- | --- | --- | --- | --- | --- | --- |
| **HRU per 1,000 patients** | **Year 5 Pre-index** | | | | **Post-index** | | | |
|  | **Q1** | **Q2** | **Q3** | **Q4** | **Q1** | **Q2** | **Q3** | **Q4** |
| **All-cause outpatient services** |  |  |  |  |  |  |  |  |
| Physician office visits | 2,894.02 | 2,884.06 | 2,903.69 | 3,066.94 | 3,819.65 | 2,930.94 | 2,709.13 | 2,564.38 |
| Outpatient visits | 3,593.71 | 3,602.53 | 3,685.51 | 4,067.16 | 5,137.43 | 3,880.15 | 3,618.76 | 3,450.12 |
| Home health services^a^ | 192.40 | 196.32 | 207.16 | 240.59 | 363.34 | 278.45 | 271.24 | 274.97 |
| Hospice^a^ | 3.41 | 4.25 | 4.39 | 3.88 | 10.18 | 18.60 | 25.89 | 35.67 |
| Nursing facility residency^a^ | 40.17 | 46.43 | 59.69 | 88.66 | 155.85 | 141.42 | 152.45 | 166.65 |
| **All-cause inpatient services** |  |  |  |  |  |  |  |  |
| Inpatient admissions | 68.92 | 75.36 | 87.63 | 179.58 | 256.96 | 104.73 | 97.59 | 109.67 |
| Emergency department visits | 252.05 | 279.11 | 324.59 | 590.34 | 728.50 | 377.91 | 366.85 | 398.31 |
| Skilled nursing facility admissions | 18.86 | 20.29 | 25.41 | 52.15 | 89.17 | 28.05 | 27.17 | 32.30 |
| **Non-AD cohort (N = 27,308)** | | | | | | | | |
| **HRU per 1,000 patients** | **Year 5 Pre-index** | | | | **Post-index** | | | |
|  | **Q1** | **Q2** | **Q3** | **Q4** | **Q1** | **Q2** | **Q3** | **Q4** |
| **All-cause outpatient services** |  |  |  |  |  |  |  |  |
| Physician office visits | 3,057.05 | 2,935.15 | 2,984.91 | 2,173.83 | 3,919.51 | 3,131.68 | 3,148.78 | 2,901.16 |
| Outpatient visits | 3,641.86 | 3,527.79 | 3,581.22 | 2,679.32 | 4,720.30 | 3,839.57 | 3,842.21 | 3,568.30 |
| Home health services^a^ | 140.73 | 132.85 | 130.80 | 119.12 | 184.41 | 154.06 | 151.09 | 156.69 |
| Hospice^a^ | 0.59 | 0.66 | 0.70 | 0.88 | 1.17 | 2.12 | 3.55 | 6.63 |
| Nursing facility residency^a^ | 4.98 | 5.24 | 5.79 | 5.64 | 8.83 | 7.95 | 9.78 | 9.78 |
| **All-cause Inpatient Services** |  |  |  |  |  |  |  |  |
| Inpatient admissions | 39.84 | 39.07 | 37.35 | 29.52 | 64.78 | 45.52 | 44.82 | 51.71 |
| Emergency department visits | 124.98 | 121.65 | 119.82 | 92.21 | 217.56 | 149.99 | 145.93 | 169.91 |
| Skilled nursing facility admissions | 6.30 | 7.21 | 6.23 | 5.38 | 11.21 | 8.72 | 9.04 | 9.48 |

Unless otherwise stated, “HRU” reflects the number of events per 1,000 patients.

^a^Rates for home health services, hospice, and nursing facility residency reflect the number of patients per 1,000 (ie, proportion of patients).

**Abbreviations:** AD, Alzheimer’s disease; HRU, healthcare resource use.

**Table C.1.** Pre- and post-diagnosis patterns of healthcare resource use associated with AD dementia and AD dementia-related complications for matched AD dementia and non-AD cohorts.

| **AD dementia cohort (N = 27,308)** | | | | | | | | |
| --- | --- | --- | --- | --- | --- | --- | --- | --- |
| **HRU per 1,000 patients** | **Year 1 Pre-index** | | | | **Year 2 Pre-index** | | | |
|  | **Q1** | **Q2** | **Q3** | **Q4** | **Q1** | **Q2** | **Q3** | **Q4** |
| **AD-related** |  |  |  |  |  |  |  |  |
| Physician office visits | 20.58 | 24.02 | 25.56 | 27.13 | 30.83 | 38.63 | 39.44 | 40.76 |
| Emergency department visits | 3.22 | 2.93 | 2.56 | 3.59 | 3.55 | 6.01 | 5.13 | 5.86 |
| Inpatient admissions | 0.37 | 0.11 | 0.33 | 0.33 | 0.33 | 0.55 | 0.15 | 0.62 |
| **Skin ulcer-related** |  |  |  |  |  |  |  |  |
| Physician office visits | 22.89 | 21.72 | 23.11 | 25.60 | 26.04 | 25.34 | 22.81 | 25.19 |
| Emergency department visits | 2.34 | 1.94 | 2.16 | 1.98 | 1.76 | 2.27 | 2.23 | 2.78 |
| Inpatient admissions | 0.81 | 0.73 | 0.62 | 0.55 | 0.55 | 0.55 | 0.77 | 0.81 |
| **UTI-related** |  |  |  |  |  |  |  |  |
| Physician office visits | 26.26 | 27.57 | 26.73 | 28.60 | 25.08 | 26.84 | 26.73 | 28.09 |
| Emergency department visits | 4.80 | 4.36 | 4.80 | 5.31 | 5.16 | 5.90 | 5.64 | 7.21 |
| Inpatient admissions | 1.21 | 1.14 | 1.17 | 1.35 | 1.68 | 1.25 | 1.25 | 2.23 |
| **Fall/fracture-related** |  |  |  |  |  |  |  |  |
| Physician office visits | 36.55 | 38.78 | 41.86 | 35.63 | 35.01 | 36.88 | 30.47 | 30.98 |
| Emergency department visits | 7.32 | 7.10 | 8.53 | 9.01 | 7.47 | 8.09 | 9.19 | 9.08 |
| Inpatient admissions | 2.49 | 2.71 | 3.00 | 2.82 | 2.38 | 3.30 | 2.93 | 3.30 |
| **Malnutrition-related** |  |  |  |  |  |  |  |  |
| Physician office visits | 14.54 | 17.50 | 16.22 | 17.28 | 18.75 | 16.84 | 19.37 | 19.44 |
| Emergency department visits | 0.07 | 0.00 | 0.00 | 0.11 | 0.04 | 0.00 | 0.04 | 0.00 |
| Inpatient admissions | 0.00 | 0.00 | 0.00 | 0.07 | 0.04 | 0.07 | 0.04 | 0.00 |
| **Pneumonia-related** |  |  |  |  |  |  |  |  |
| Physician office visits | 4.69 | 4.94 | 5.24 | 5.49 | 5.75 | 4.69 | 4.03 | 4.36 |
| Emergency department visits | 2.23 | 2.75 | 2.16 | 3.15 | 2.16 | 2.78 | 2.60 | 3.70 |
| Inpatient admissions | 1.57 | 1.46 | 1.46 | 1.90 | 1.43 | 1.39 | 1.35 | 1.65 |

“HRU” reflects the number of events per 1,000 patients.

**Abbreviations:** AD, Alzheimer’s disease; HRU, healthcare resource use.

**Table C.1.** Pre- and post-diagnosis patterns of healthcare resource use associated with AD dementia and AD-related complications for matched AD dementia and non-AD cohorts (cont’d).

| **Non-AD cohort (N = 27,308)** | | | | | | | | |
| --- | --- | --- | --- | --- | --- | --- | --- | --- |
| **HRU per 1,000 patients** | **Year 1 Pre-index** | | | | **Year 2 Pre-index** | | | |
|  | **Q1** | **Q2** | **Q3** | **Q4** | **Q1** | **Q2** | **Q3** | **Q4** |
| **AD-related** |  |  |  |  |  |  |  |  |
| Physician office visits | 0.00 | 0.00 | 0.00 | 0.00 | 0.00 | 0.00 | 0.00 | 0.00 |
| Emergency department visits | 0.00 | 0.00 | 0.00 | 0.00 | 0.00 | 0.00 | 0.00 | 0.00 |
| Inpatient admissions | 0.00 | 0.00 | 0.00 | 0.00 | 0.00 | 0.00 | 0.00 | 0.00 |
| **Skin ulcer-related** |  |  |  |  |  |  |  |  |
| Physician office visits | 21.09 | 20.40 | 21.61 | 19.41 | 26.33 | 21.17 | 22.04 | 19.96 |
| Emergency department visits | 1.61 | 2.49 | 1.79 | 1.98 | 1.83 | 1.79 | 1.32 | 1.28 |
| Inpatient admissions | 0.51 | 0.55 | 0.48 | 0.44 | 0.59 | 0.37 | 0.29 | 0.33 |
| **UTI-related** |  |  |  |  |  |  |  |  |
| Physician office visits | 26.26 | 24.32 | 30.50 | 22.52 | 25.05 | 25.89 | 26.26 | 22.48 |
| Emergency department visits | 3.59 | 3.44 | 3.99 | 3.81 | 2.75 | 3.22 | 2.53 | 2.49 |
| Inpatient admissions | 0.88 | 0.84 | 0.73 | 0.99 | 0.33 | 0.55 | 0.62 | 0.62 |
| **Fall/fracture-related** |  |  |  |  |  |  |  |  |
| Physician office visits | 33.40 | 34.42 | 30.58 | 33.80 | 35.41 | 30.47 | 27.83 | 25.78 |
| Emergency department visits | 6.04 | 5.35 | 5.57 | 7.62 | 4.91 | 5.49 | 5.53 | 5.90 |
| Inpatient admissions | 1.90 | 2.01 | 1.76 | 2.05 | 1.57 | 2.16 | 1.57 | 1.76 |
| **Malnutrition-related** |  |  |  |  |  |  |  |  |
| Physician office visits | 13.07 | 13.59 | 13.48 | 14.35 | 14.32 | 16.41 | 16.15 | 14.90 |
| Emergency department visits | 0.04 | 0.00 | 0.00 | 0.07 | 0.00 | 0.00 | 0.00 | 0.00 |
| Inpatient admissions | 0.04 | 0.00 | 0.00 | 0.00 | 0.00 | 0.00 | 0.00 | 0.00 |
| **Pneumonia-related** |  |  |  |  |  |  |  |  |
| Physician office visits | 5.71 | 4.39 | 4.47 | 6.12 | 6.23 | 4.65 | 4.17 | 5.49 |
| Emergency department visits | 2.34 | 1.79 | 2.27 | 3.30 | 1.72 | 1.87 | 1.98 | 2.09 |
| Inpatient admissions | 1.54 | 1.43 | 1.06 | 1.61 | 0.88 | 0.95 | 0.81 | 1.06 |

“HRU” reflects the number of events per 1,000 patients.

**Abbreviations:** AD, Alzheimer’s disease; HRU, healthcare resource use.

**Table C.1.** Pre- and post-diagnosis patterns of healthcare resource use associated with AD dementia and AD dementia-related complications for matched AD dementia and non-AD cohorts (cont’d).

| **AD dementia cohort (N = 27,308)** | | | | | | | | |
| --- | --- | --- | --- | --- | --- | --- | --- | --- |
| **HRU per 1,000 Patients** | **Year 3 Pre-index** | | | | **Year 4 Pre-index** | | | |
|  | **Q1** | **Q2** | **Q3** | **Q4** | **Q1** | **Q2** | **Q3** | **Q4** |
| **AD-related** |  |  |  |  |  |  |  |  |
| Physician office visits | 47.20 | 54.67 | 60.09 | 66.83 | 77.19 | 85.40 | 94.48 | 104.91 |
| Emergency department visits | 6.12 | 7.03 | 6.70 | 7.03 | 7.91 | 9.08 | 11.46 | 13.81 |
| Inpatient admissions | 0.55 | 0.62 | 0.66 | 0.66 | 0.81 | 0.77 | 1.25 | 1.39 |
| **Skin ulcer-related** |  |  |  |  |  |  |  |  |
| Physician office visits | 23.29 | 24.79 | 25.82 | 23.66 | 27.13 | 27.32 | 24.61 | 22.63 |
| Emergency department visits | 3.00 | 2.56 | 2.71 | 3.59 | 3.52 | 3.59 | 3.30 | 4.39 |
| Inpatient admissions | 0.92 | 0.73 | 0.88 | 1.06 | 0.84 | 1.21 | 0.99 | 1.32 |
| **UTI-related** |  |  |  |  |  |  |  |  |
| Physician office visits | 26.51 | 28.86 | 28.56 | 28.71 | 28.78 | 27.79 | 30.69 | 32.81 |
| Emergency department visits | 5.68 | 8.83 | 7.54 | 7.80 | 8.13 | 9.74 | 11.54 | 12.85 |
| Inpatient admissions | 1.25 | 1.79 | 1.54 | 1.72 | 2.12 | 2.16 | 2.82 | 2.45 |
| **Fall/fracture-related** |  |  |  |  |  |  |  |  |
| Physician office visits | 37.32 | 32.99 | 36.11 | 39.04 | 35.56 | 34.61 | 34.17 | 30.61 |
| Emergency department visits | 10.33 | 10.55 | 12.19 | 11.35 | 11.79 | 13.92 | 13.88 | 14.87 |
| Inpatient admissions | 3.77 | 3.66 | 4.69 | 4.14 | 3.22 | 4.58 | 4.36 | 4.61 |
| **Malnutrition-related** |  |  |  |  |  |  |  |  |
| Physician office visits | 20.95 | 19.48 | 21.28 | 21.06 | 21.02 | 21.93 | 22.30 | 22.63 |
| Emergency department visits | 0.00 | 0.00 | 0.04 | 0.07 | 0.04 | 0.00 | 0.00 | 0.07 |
| Inpatient admissions | 0.00 | 0.00 | 0.04 | 0.04 | 0.07 | 0.00 | 0.00 | 0.04 |
| **Pneumonia-related** |  |  |  |  |  |  |  |  |
| Physician office visits | 4.65 | 6.08 | 5.49 | 4.87 | 3.95 | 5.71 | 5.42 | 6.04 |
| Emergency department visits | 3.66 | 3.88 | 4.06 | 4.03 | 3.00 | 4.83 | 5.16 | 5.13 |
| Inpatient admissions | 1.90 | 2.31 | 1.72 | 1.87 | 1.32 | 2.71 | 2.60 | 2.67 |

“HRU” reflects the number of events per 1,000 patients.

**Abbreviations:** AD, Alzheimer’s disease; HRU, healthcare resource use; UTI, urinary tract infection.

**Table C.1.** Pre- and post-diagnosis patterns of healthcare resource use associated with AD dementia and AD dementia-related complications for matched AD dementia and non-AD cohorts (cont’d).

| **Non-AD cohort (N = 27,308)** | | | | | | | | |
| --- | --- | --- | --- | --- | --- | --- | --- | --- |
| **HRU per 1,000 patients** | **Year 3 Pre-index** | | | | **Year 4 Pre-index** | | | |
|  | **Q1** | **Q2** | **Q3** | **Q4** | **Q1** | **Q2** | **Q3** | **Q4** |
| **AD-related** |  |  |  |  |  |  |  |  |
| Physician office visits | 0.00 | 0.00 | 0.00 | 0.00 | 0.00 | 0.00 | 0.00 | 0.00 |
| Emergency department visits | 0.00 | 0.00 | 0.00 | 0.00 | 0.00 | 0.00 | 0.00 | 0.00 |
| Inpatient admissions | 0.00 | 0.00 | 0.00 | 0.00 | 0.00 | 0.00 | 0.00 | 0.00 |
| **Skin ulcer-related** |  |  |  |  |  |  |  |  |
| Physician office visits | 23.80 | 23.88 | 24.72 | 25.12 | 27.76 | 22.89 | 26.29 | 23.00 |
| Emergency department visits | 2.20 | 2.16 | 2.31 | 1.65 | 2.23 | 2.31 | 2.42 | 2.27 |
| Inpatient admissions | 0.59 | 0.70 | 0.51 | 0.66 | 0.70 | 0.62 | 0.66 | 0.62 |
| **UTI-related** |  |  |  |  |  |  |  |  |
| Physician office visits | 22.12 | 25.30 | 27.65 | 24.50 | 26.33 | 24.50 | 27.21 | 22.78 |
| Emergency department visits | 2.93 | 3.08 | 3.08 | 3.19 | 3.66 | 3.74 | 4.32 | 3.26 |
| Inpatient admissions | 0.55 | 0.44 | 0.40 | 0.70 | 0.51 | 0.70 | 0.51 | 0.70 |
| **Fall/fracture-related** |  |  |  |  |  |  |  |  |
| Physician office visits | 28.89 | 31.13 | 30.17 | 35.81 | 33.54 | 30.76 | 26.51 | 27.24 |
| Emergency department visits | 5.38 | 5.68 | 5.68 | 7.25 | 5.79 | 7.03 | 5.86 | 7.29 |
| Inpatient admissions | 2.12 | 1.50 | 2.05 | 3.08 | 2.12 | 2.45 | 1.83 | 2.34 |
| **Malnutrition-related** |  |  |  |  |  |  |  |  |
| Physician office visits | 15.27 | 16.70 | 16.04 | 17.39 | 19.08 | 18.35 | 18.20 | 19.12 |
| Emergency department visits | 0.04 | 0.00 | 0.00 | 0.00 | 0.00 | 0.04 | 0.18 | 0.07 |
| Inpatient admissions | 0.04 | 0.00 | 0.00 | 0.00 | 0.00 | 0.04 | 0.00 | 0.00 |
| **Pneumonia-related** |  |  |  |  |  |  |  |  |
| Physician office visits | 4.94 | 4.54 | 3.95 | 6.01 | 6.15 | 5.09 | 4.58 | 6.59 |
| Emergency department visits | 2.53 | 2.45 | 2.01 | 2.82 | 3.30 | 2.42 | 2.05 | 3.59 |
| Inpatient admissions | 1.25 | 1.25 | 0.81 | 1.39 | 1.50 | 1.39 | 1.46 | 1.65 |

“HRU” reflects the number of events per 1,000 patients.

**Abbreviations:** AD, Alzheimer’s disease; HRU, healthcare resource use; UTI, urinary tract infection.

**Table C.1.** Pre- and post-diagnosis patterns of healthcare resource use associated with AD dementia and AD dementia-related complications for matched AD dementia and non-AD cohorts (cont’d).

| **AD dementia cohort (N = 27,308)** | | | | | | | | |
| --- | --- | --- | --- | --- | --- | --- | --- | --- |
| **HRU per 1,000 patients** | **Year 5 Pre-index** | | | | **Post-index** | | | |
|  | **Q1** | **Q2** | **Q3** | **Q4** | **Q1** | **Q2** | **Q3** | **Q4** |
| **AD-related** |  |  |  |  |  |  |  |  |
| Physician office visits | 115.50 | 137.36 | 164.82 | 278.60 | 728.47 | 298.41 | 245.75 | 229.20 |
| Emergency department visits | 14.32 | 18.38 | 27.13 | 83.71 | 118.68 | 42.99 | 40.68 | 44.68 |
| Inpatient admissions | 1.39 | 1.98 | 2.89 | 10.55 | 38.08 | 8.06 | 6.08 | 7.43 |
| **Skin Ulcer-related** |  |  |  |  |  |  |  |  |
| Physician office visits | 26.48 | 28.34 | 28.78 | 26.59 | 34.50 | 31.60 | 31.60 | 26.99 |
| Emergency department visits | 4.69 | 4.25 | 5.02 | 7.47 | 10.00 | 6.01 | 5.93 | 6.44 |
| Inpatient admissions | 1.25 | 1.10 | 1.10 | 2.60 | 3.70 | 1.83 | 1.68 | 1.72 |
| **UTI-related** |  |  |  |  |  |  |  |  |
| Physician office visits | 31.27 | 32.70 | 33.73 | 37.94 | 46.84 | 36.91 | 34.82 | 33.07 |
| Emergency department visits | 12.93 | 14.14 | 19.48 | 39.26 | 45.44 | 25.63 | 25.19 | 29.04 |
| Inpatient admissions | 3.00 | 3.00 | 4.43 | 10.73 | 13.55 | 6.34 | 4.83 | 6.66 |
| **Fall/fracture-related** |  |  |  |  |  |  |  |  |
| Physician office visits | 37.86 | 40.39 | 45.37 | 51.82 | 74.08 | 47.46 | 38.12 | 39.51 |
| Emergency department visits | 17.50 | 17.76 | 21.83 | 45.19 | 44.16 | 25.45 | 24.13 | 30.03 |
| Inpatient admissions | 5.71 | 5.79 | 8.06 | 18.20 | 18.68 | 8.20 | 8.39 | 9.01 |
| **Malnutrition-related** |  |  |  |  |  |  |  |  |
| Physician office visits | 22.92 | 22.23 | 23.07 | 24.35 | 29.99 | 22.34 | 19.37 | 19.85 |
| Emergency department visits | 0.04 | 0.07 | 0.00 | 0.11 | 0.11 | 0.07 | 0.22 | 0.15 |
| Inpatient admissions | 0.04 | 0.04 | 0.00 | 0.11 | 0.15 | 0.04 | 0.04 | 0.07 |
| **Pneumonia-related** |  |  |  |  |  |  |  |  |
| Physician office visits | 6.34 | 5.64 | 5.35 | 6.48 | 11.64 | 6.19 | 7.62 | 7.51 |
| Emergency department visits | 5.68 | 5.79 | 6.04 | 13.55 | 18.46 | 8.72 | 10.40 | 12.30 |
| Inpatient admissions | 2.64 | 2.78 | 3.33 | 5.82 | 9.70 | 3.59 | 4.54 | 5.49 |

“HRU” reflects the number of events per 1,000 patients.

**Abbreviations:** AD, Alzheimer’s disease; HRU, healthcare resource use; UTI, urinary tract infection.

**Table C-1.** Pre- and post-diagnosis patterns of healthcare resource use associated with AD dementia and AD dementia-related complications for matched AD dementia and non-AD cohorts (cont’d).

| **Non-AD cohort (N = 27,308)** | | | | | | | | |
| --- | --- | --- | --- | --- | --- | --- | --- | --- |
| **HRU per 1,000 patients** | **Year 5 Pre-index** | | | | **Post-index** | | | |
|  | **Q1** | **Q2** | **Q3** | **Q4** | **Q1** | **Q2** | **Q3** | **Q4** |
| **AD-related** |  |  |  |  |  |  |  |  |
| Physician office visits | 0.00 | 0.00 | 0.00 | 0.00 | 0.00 | 0.00 | 0.00 | 0.00 |
| Emergency department visits | 0.00 | 0.00 | 0.00 | 0.00 | 0.00 | 0.00 | 0.00 | 0.00 |
| Inpatient admissions | 0.00 | 0.00 | 0.00 | 0.00 | 0.00 | 0.00 | 0.00 | 0.00 |
| **Skin ulcer-related** |  |  |  |  |  |  |  |  |
| Physician office visits | 27.13 | 24.79 | 21.02 | 14.68 | 33.95 | 29.73 | 27.83 | 25.60 |
| Emergency department visits | 2.67 | 3.33 | 2.38 | 1.57 | 3.81 | 2.97 | 2.67 | 2.75 |
| Inpatient admissions | 0.62 | 0.77 | 0.95 | 0.26 | 1.03 | 0.99 | 0.70 | 0.88 |
| **UTI-related** |  |  |  |  |  |  |  |  |
| Physician office visits | 22.92 | 26.88 | 25.74 | 17.80 | 33.43 | 26.18 | 26.73 | 24.61 |
| Emergency department visits | 4.10 | 4.91 | 4.61 | 3.52 | 7.51 | 5.24 | 6.30 | 6.48 |
| Inpatient admissions | 0.66 | 0.51 | 0.70 | 0.40 | 1.35 | 0.66 | 0.66 | 1.25 |
| **Fall/fracture-related** |  |  |  |  |  |  |  |  |
| Physician office visits | 31.46 | 30.83 | 28.60 | 25.78 | 46.95 | 36.69 | 31.31 | 27.87 |
| Emergency department visits | 5.68 | 5.82 | 6.34 | 5.71 | 13.73 | 8.35 | 9.34 | 10.40 |
| Inpatient admissions | 2.01 | 1.98 | 2.34 | 1.65 | 4.17 | 2.12 | 2.75 | 3.30 |
| **Malnutrition-related** |  |  |  |  |  |  |  |  |
| Physician office visits | 19.66 | 19.41 | 19.52 | 15.38 | 22.96 | 22.85 | 21.75 | 18.53 |
| Emergency department visits | 0.00 | 0.00 | 0.00 | 0.00 | 0.18 | 0.00 | 0.00 | 0.00 |
| Inpatient admissions | 0.00 | 0.00 | 0.00 | 0.00 | 0.00 | 0.00 | 0.00 | 0.00 |
| **Pneumonia-related** |  |  |  |  |  |  |  |  |
| Physician office visits | 7.95 | 5.13 | 4.32 | 4.8 | 11.1 | 6.26 | 5.27 | 6.99 |
| Emergency department visits | 4.54 | 2.20 | 3.00 | 3.11 | 5.79 | 3.95 | 3.55 | 5.64 |
| Inpatient admissions | 2.42 | 1.25 | 1.65 | 1.61 | 2.53 | 2.01 | 1.61 | 2.60 |

“HRU” reflects the number of events per 1,000 patients.

**Abbreviations:** AD, Alzheimer’s disease; HRU, healthcare resource use; UTI, urinary tract infection.
